# Graphene ballistic nano-rectifier with very high responsivity

**DOI:** 10.1038/ncomms11670

**Published:** 2016-05-31

**Authors:** Gregory Auton, Jiawei Zhang, Roshan Krishna Kumar, Hanbin Wang, Xijian Zhang, Qingpu Wang, Ernie Hill, Aimin Song

**Affiliations:** 1 School of Electrical and Electronic Engineering, University of Manchester, Manchester M13 9PL, UK; 2 Manchester Centre for Mesoscience and Nanotechnology, University of Manchester, Manchester M13 9PL, UK; 3 School of Physics and Astronomy, University of Manchester, M13 9PL Manchester, UK; 4 School of Physics and Center of Nanoelectronics, Shandong University, Jinan 250100, China

## Abstract

Although graphene has the longest mean free path of carriers of any known electronic material, very few novel devices have been reported to harness this extraordinary property. Here we demonstrate a ballistic nano-rectifier fabricated by creating an asymmetric cross-junction in single-layer graphene sandwiched between boron nitride flakes. A mobility ∼200,000 cm^2^ V^−1^ s^−1^ is achieved at room temperature, well beyond that required for ballistic transport. This enables a voltage responsivity as high as 23,000 mV mW^−1^ with a low-frequency input signal. Taking advantage of the output channels being orthogonal to the input terminals, the noise is found to be not strongly influenced by the input. Hence, the corresponding noise-equivalent power is as low as 0.64 pW Hz^−1/2^. Such performance is even comparable to superconducting bolometers, which however need to operate at cryogenic temperatures. Furthermore, output oscillations are observed at low temperatures, the period of which agrees with the lateral size quantization.

## Abstract (web summary)

The high mobility of graphene has the potential to enable design of devices working in a collisionless regime of carrier transport. Here the authors fabricate a ballistic nano-rectifier based on encapsulated graphene, showing intrinsic performances comparable to those of superconducting bolometers.

## Graphical abstract

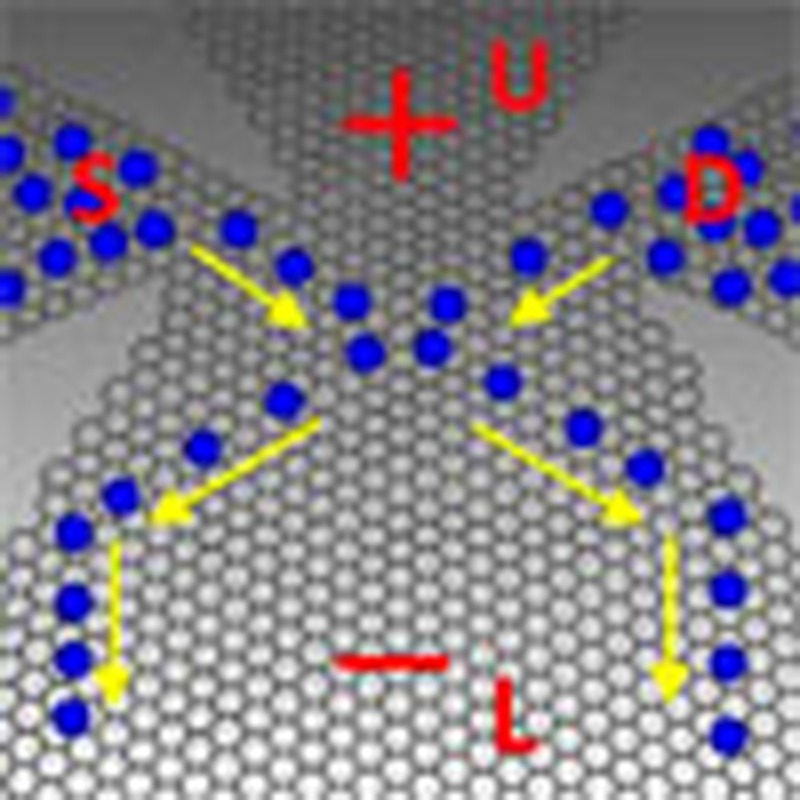

Recent developments in graphene device fabrication techniques, most notably the introduction of stamp transfer and one-dimensional contacts, have led to carrier mobilities >100,000 cm^2^ V^−1^ s^−1^ at room temperature^1^. This exceptional property enables graphene to possess a carrier mean free path (*λ*_MFP_) longer than 1 μm at room temperature. Despite this, even the most promising applications to commercialize graphene in the electronics industry do not take advantage of this property. Instead, they often attempt to tackle the problem that graphene has no bandgap by using, for example, vertical tunnelling transistors^2^ and graphene nano-ribbons^3^. To bring a device into the ballistic regime of carrier transport, the device architecture needs to be planar and the device active area must be smaller than *λ*_MFP_. This means that the carriers would be more likely to scatter off the edges in the device than off randomly distributed phonons or defects. Hence, a simplistic billiard ball model could be used for the transport of carriers through the device. One such planar, ballistic device that can take advantage of graphene is the ballistic rectifier (BR), which is an asymmetric four-terminal cross-junction that relies on the ‘billiard ball' motion of carriers to rectify a.c. signals into d.c. outputs^4^. The BR was first demonstrated in InGaAs/AlGaAs hetero-structures and its full-wave rectifying functionality resembles that of a bridge rectifier, which in contrast requires four individual diodes. Similar asymmetric three-terminal junctions were also studied recently^5^,^6^. The BR concept inherently differs from all conventional rectifiers because it neither contains nor relies on any doping junction or barrier structure along the direction of electrical current. As such, the carriers do not need to overcome a built-in electric field to conduct a current, meaning that the device has a zero threshold voltage. This is important at high frequency because the signals are often very small and can even be below the threshold of a conventional diode. Furthermore, unlike conventional diodes or transistors, the new working principle does not require a material with an energy bandgap and the device speed scales up with the carrier mobility. Hence, graphene, despite having a zero bandgap, is ideal for the fabrication of BRs. One of the most promising features of the BR is that it is planar that results in low parasitic capacitance and hence a high cut-off frequency^7^. The device can be used in conjunction with an antenna in security imaging, biomedical diagnostics, optical heterodyne detection, thermo-electric energy harvesting and cooling^8^,^9^. Very recently, ballistic rectification was demonstrated in graphene^10^. However, the substrate used was SiO_2_, which leaves charge puddles in the graphene that scatter the carriers and lower the mobility to about 1,810 cm^2^ V^−1^ s^−1^, making the device not truly in the ballistic regime. This resulted in a responsivity of 110 mV mW^−1^ and a noise-equivalent power (NEP) of 10^−9^ W Hz^−1/2^ (Supplementary Equations 1 and 2). Such a performance matched commercially available uncooled bolometers, but did not truly take advantage of graphene's high mobility. Here, to enable the BR truly in the ballistic transport regime, hexagonal boron nitride (h-BN) is used as the substrate instead. Since, h-BN has very few dangling bonds and an atomically flat surface, it is an excellent substrate for graphene^11^. We are able to achieve mobility close to the theoretical limit up to nearly 200,000 cm^2^ V^−1^ s^−1^, leading to a marked improvement of the responsivity as high as 23,000 mV mW^−1^ and an NEP as low as 0.64 pW Hz^−1/2^ at room temperature. Such a performance has thus far only been matched by superconducting bolometers, which however must operate at cryogenic temperatures.

## Results

### Device design and graphene characterization

**Figure 1:**
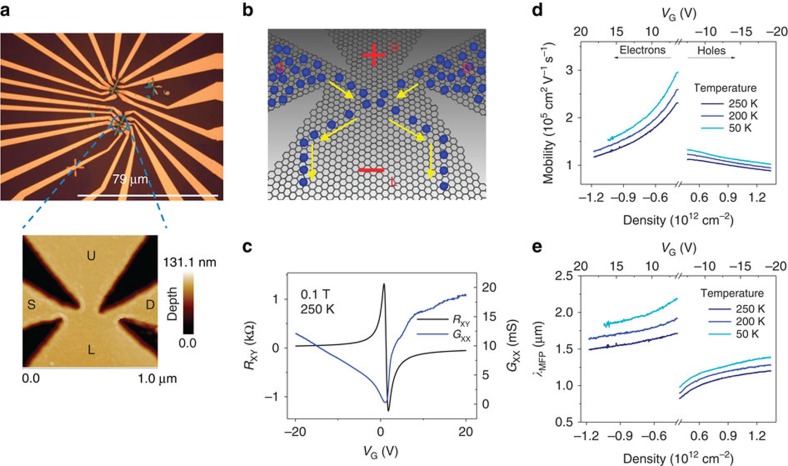
Initial geometric characterization of the device and the electrical properties of the graphene. (**a**) × 100 magnification optical image of a typical device with the ballistic rectifier (BR) identified by the blue circle. An atomic-force micrograph of the active region of the circled device has been included to show the geometry of the BR. The yellow regions are where the graphene is masked during the etching step. (**b**) A schematic representation of a BR where typical carrier paths are shown by the yellow arrows, demonstrating the preferred transmission of ballistic charge carriers to the lower terminal (L) with respect to the upper (U) terminal when the source (S) and drain (D) contacts are connected to an a.c. input. (**c**) Hall resistance and longitudinal conductance of an empty segment of the Hall bar without BR as a function of back-gate voltage at 250 K with an applied magnetic field of 0.1 T. (**d**,**e**) Obtained mobility from the Hall measurements and the calculated mean free path for electrons and holes as a function of carrier density, respectively, at different temperatures of 50, 200 and 250 K. The arrows in **d** indicate the direction of increasing carrier concentration of each carrier type.

Figure 1a shows a 100 × optical image and an atomic-force micrograph of the BR active region of a typical device on a hall bar. Figure 1b is a schematic diagram of a BR showing typical carrier paths by the blue arrows, demonstrating preferred transmission of ballistic charge carriers to the lower terminal (L) with respect to the upper (U) terminal when the source (S) and drain (D) contacts are connected to an a.c. input. The planar nature of this rectifier takes advantage of the high carrier mobility and long mean free path of graphene. According to a quantitative model in ref. 12, which was on a slightly different design, the rectifying functionality is based on the collimating effect of carriers induced by the applied current. The collimating effect changes the carrier transmission probabilities from S and D to the L and U terminals, and hence according to the Büttiker–Landauer formalism induces a d.c. voltage difference between L and U^12^. Such a rectifying effect therefore does not rely on using any doping junction or Schottky barrier as in the case of a conventional diode, but the device needs to operate in the ballistic transport regime and hence typically at the nanoscale.

Figure 1c shows both the Hall resistance *R*_XY_ and the longitudinal conductance *G*_XX_ when a magnetic field of 0.1 T is applied at 250 K, as a function of the back-gate voltage *V*_G_. Figure 1d,e shows the calculated mobility and mean free path *λ*_MFP_, respectively, from the Hall data at 50, 200 and 250 K as a function of the carrier density. The region close to the Dirac point is excluded because of the existence of both carriers due to thermal excitation. The mean free path is calculated from 
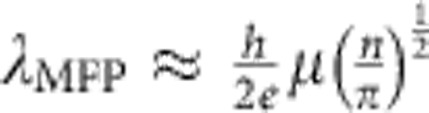
 (ref. 13. Both the electrons (indicated by negative density) and holes increase their mean free path at lower temperatures. The carrier mobilities have also been determined using the electrostatic gating method at room temperature (shown in Supplementary Fig. 1), giving values of 197,600 cm^2^ V^−1^ s^−1^ and 106,000 cm^2^ V^−1^s^−1^ for electrons and holes, respectively. This is in good agreement with the Hall measurements and ensures that the device in Fig. 1a operates in the ballistic transport regime.

### Device characterization

**Figure 2:**
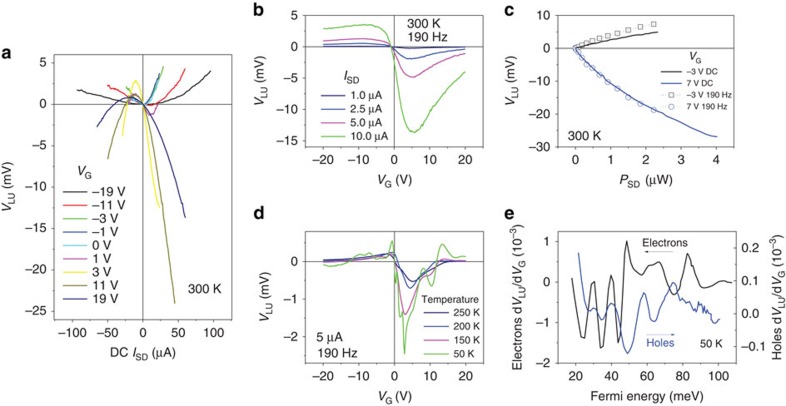
Detailed electrical characterization. (**a**) d.c. output voltage between L and U, *V*_LU_, as a function of d.c. current through S and D, *I*_SD_, at different gate voltages, *V*_G_, for a BR at room temperature. (**b**) *V*_LU_ as a function of *V*_G_ at four different input a.c.'s at room temperature. (**c**) Dependence of the average d.c. output *V*_LU_ on the input d.c. and a.c. power *P*_SD_, showing good agreement. (**d**) *V*_LU_ as a function of *V*_G_ for a low input a.c. current of *I*_SD_=1 μA at 190 Hz at four different temperatures. (**e**) Differential of *V*_LU_ with respect to *V*_G_ as a function of the Fermi energy for both electrons and holes at 50 K.

Figure 2a shows the output voltage between L and U, *V*_LU_, as a function of d.c. current through S and D, *I*_SD_, at different gate voltages for a BR at room temperature. The neutrality point is at *V*_G_=−1 V. The change from positive to negative curvature in the curves with the switch of the carrier type demonstrates the ambipolar nature of the device. The result also shows that the device operates optimally close to (but not exactly at) the neutrality point. It is useful to first check if the voltage output from these devices could be a result of the Seebeck effect. The Seebeck effect describes the process of hot carriers, travelling towards cool regions and it is one of the origins of thermo-electric effect. When the current is passed from S to D, the active region of the device will heat up due to carrier interactions with the lattice. In turn, the asymmetric nature of the device may create a temperature gradient in the vertical direction of the cross-junction and therefore a net voltage difference. This effect has been studied in graphene and other materials, and does produce a quadratic response^13^,^14^. For n-type (or p-type) carrier transport, the Seebeck effect is expected to generate a positive (or negative) *V*_LU_, due to the asymmetric nature of the device. We have observed that the output is in fact negative (or positive) *V*_LU_. Therefore, any thermal rectification effect that may be present in our devices is at least less dominant than the ballistic rectifying effect at room temperature.

Figure 2b shows the d.c.-rectified output, as a function of the gate voltage for different a.c. input currents at 190 Hz. The change in the sign of the d.c. output also occurs around the Dirac point. It is now easy to see that the device operates optimally around *V*_G_=+5 V for the electrons and around *V*_G_=−8 V for the holes. The output further away from the neutrality point becomes lower, which is a direct result of increased carrier density and Fermi energy^12^. As the carrier density increases, the conductivity of the cross-junction increases (the variation in the source-drain resistance is show in Supplementary Fig. 2). The increased momentum of the individual carriers makes them harder to collimate along the applied current direction, hence causing reduced output^12^,^13^. On the other hand, when the device is gate-biased too close to the Dirac point, there is a coexistence of both types of carriers due to thermal excitation, and the electrons and holes produce opposite outputs resulting in a decreased *V*_LU_. Furthermore, the output is generally stronger in the electron transport region than for holes, which reflects the different carrier mobilities. Figure 2c shows the output voltage response to both d.c. and a.c. input power, *P*_SD_, which are in good agreement. The dependence is quite linear at low input power, as expected from the extended Büttiker–Landauer formalism^12^. The deviation from the linear dependence at high power is expected in ref. 10 and could partially be a result of the current density going as *J*∝*E*^α^ in the non-linear regime in graphene, where 1>*α*>1.5 and *E* is the electric field strength^15^,^16^.

Figure 2d demonstrates *V*_LU_ dependence on *V*_G_ at a low-a.c. input of 1 μA at different temperatures. It can be seen that the device output in general increases as the temperature decreases, since the value of *λ*_MFP_ is larger at lower temperatures. When electrons are the majority carriers, the output shows more dependence on temperature than in the hole transport region, which may be a result of the different dependences in carrier mobility and mean free path with temperature as shown in Fig. 1d. In the lower-temperature cases, the output even reverses, for example, as *V*_G_ is higher than +13 V or lower than −12 V. Both the Seebeck effect and ballistic rectifying effect are expected to present in the device, and the latter is known to weaken at high-gate voltages because of high carrier concentrations. So, the output reversal is likely due to the Seebeck effect that becomes more dominant at large gate biases^14^,^17^.

One prominent feature that the ballistic model does not explain is the oscillations at 50 K. They may look like mesoscopic conductance fluctuations at first glance, but they are observed also at 150 K. Conductance fluctuations are typically washed out at 5 K (ref. 18). Shubnikov–de Haas oscillations are observed at room temperature, but only in a high magnetic field that is not the case here. Electron traps and edge states may introduce output minima, but it is unlikely that these would be observed at these energies with this periodicity and magnitude of oscillation^19^,^20^,^21^. Previously, quantum oscillations have been observed at a relatively high temperatures in BRs made of a conventional semiconductor InGaAs^22^. The phenomenon was attributed to the lateral quantum confinement modes in the narrow S and D channels. By plotting d*V*_LU_/d*V*_G_ against Fermi energy (*E*_F_) in Fig. 2e, a quasi-periodic structure can be observed. The separation of the peaks (Δ*E*) fluctuates around a mean value of 15.1 meV. The lateral quantization fulfils *k*_⊥_*W*_SD_=*πm*, where *k*_⊥_ is the wave vector perpendicular to the direction of transport, *W*_SD_ is the narrowest part of the injection leads and *m* is the number of conducting modes^23^. This leads to the separation of energy modes Δ*E*=*ħv*_F_*π*/*W*_SD_, where *v*_F_ is the Fermi velocity (10^6^ ms^−1^ in graphene). The average Δ*E* for this device predicts a *W*_SD_ of 71 nm. The width is in the same order of magnitude as, but less than those measured with atomic-force micrograph, 122 nm for the narrowest region of the source lead and 137 nm for the drain. It is likely that the ballistically conducting region of the graphene does not extend all the way to the edge due to edge states and traps (Coulomb blockade and single-electron effect of graphene constrictions)^5^. We also note that the carrier concentration *n* and hence 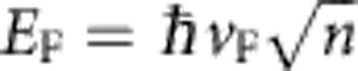 are measured independently on the Hall bar, and may not be the same as in the channel. Variation in the peak separation is likely due to the fact that the source and drain leads do not have perfectly identical widths, which may induce slightly different oscillation periods and create a complicated peak structure. Furthermore, holes and electrons are expected to give opposite signs in Fig. 2e. While this does seem to occur at higher Fermi energies, it is not the case at lower Fermi energies where the above method of determining the Fermi energy may be inaccurate due to the coexistence of both carriers.

### Sensitivity and noise measurements

**Figure 3:**
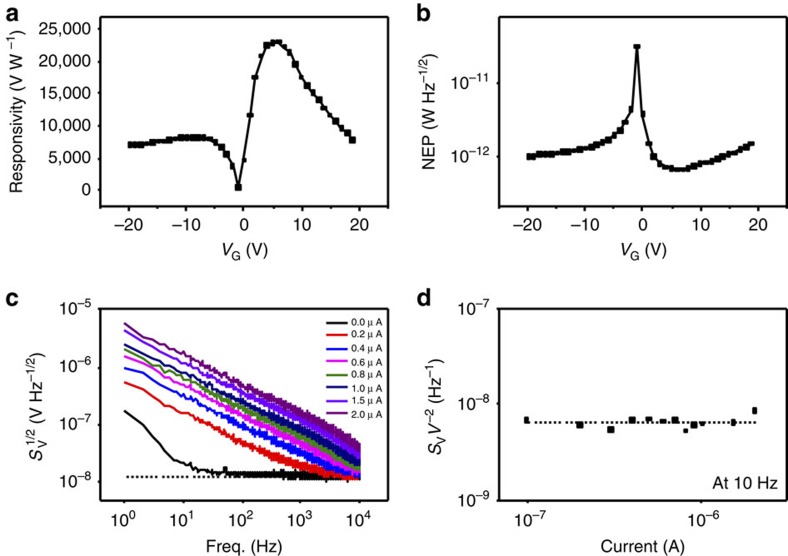
Sensitivity and noise measurements. (**a**,**b**) Responsivity and NEP of the ballistic rectifier at 190 Hz as a function of *V*_G_. (**c**) Output voltage noise spectra as a function of frequency at different input currents. (**d**) Voltage noise divided by the square of the applied bias, *S*_V_*V*_SD_^−2^, from 0.1 μA to 2.0 μA at 10 Hz, suggesting that mobility fluctuation attributes to the flicker noise at low frequencies according to Hooge's empirical equation. The dotted lines in **c** and **d** show the thermal noise level at zero input.

One of the advantages of the BR is its high speed, which was measured up to 50 GHz and simulated at terahertz (THz) frequencies even when using a conventional semiconductor InGaAs^24^. With a mobility ∼20 times higher than that in InGaAs, the graphene BR is expected to function well into the high THz frequency range, since the speed generally scales linearly with the carrier mobility. The conducting Si substrate in this work unfortunately causes a high parasitic capacitance and the device could only be measured up to 3 MHz, which agrees with the resistance-capacitance (RC) time constant of the parasitic capacitance and series resistance of the device. Devices could be tested at high frequency using a different substrates such as quartz and then a localized gate could be added to change the Fermi energy of the graphene. Nevertheless, as a broadband detector, the sensitivity of the device may still be determined at low frequencies. Figure 3a,b shows how the responsivity and NEP, respectively, change with *V*_G_ at room temperature by applying an a.c. to the device. The responsivity is determined by the output d.c. voltage divided by the input a.c. power (the a.c. voltage drop across S and D is measured simultaneously). The optimum intrinsic responsivity was measured to be 23,000 mV mW^−1^, which is to the best of our knowledge, one of the highest in diode detectors to date^9^,^25^,^26^,^27^,^28^,^29^. In comparison, the previous BR based on low-mobility graphene on SiO_2_ had a responsivity of just 67 mV mW^−1^, >300 times lower^10^. In total, seven devices have been made and they all showed a mobility higher than 100,000 cm^2^ V^−1^ s^−1^ and a responsivity ∼10,000 mV mW^−1^.

Another advantage of the BR is that the output channels U and L are placed in the perpendicular direction or orthogonal to the input current between S and D, and the output consists of only the rectified signal. In comparison, in a normal two-terminal diode, the nonlinearity in the current–voltage characteristics is on top of a large d.c. component, and the latter generates significant low-frequency noise and directly affects the output. Flicker noise (sometimes also called 1/*f* noise) and, in the case of a tunnelling diode, shot noise are both proportional to the applied current. Furthermore, the BR has an intrinsic zero threshold, and it hence does not need any biasing circuit that are not only troublesome at very high frequencies, but also may introduce significant parasitic elements. Operating at zero bias also greatly reduces the flicker noise, which often practically limits microwave detection capability of diode detectors^30^. To study the low-frequency noise properties of the graphene BR, we have adopted the cross-correlation technique to minimize the noise from the measurement set-up (measurement set-up is shown in Supplementary Fig. 3) (ref. 31). The noise as well as field-effect measurements for the carrier mobility are carried out in atmospheric conditions over a period of many weeks and the devices show no sign of degradation. The good ambient stability is due to the encapsulation of the device in BN layers. Figure 3c shows the obtained low-frequency noise spectrum of the ballistic device, *S*_V_, as a function of frequency at different input currents. At zero input, the BR generated a white noise that is measured at 13.3 nV Hz^−1/2^. As *R*_LU_ is around 15 kΩ when no gate bias was applied (*R*_LU_ is shown in Supplementary Fig. 4), this result is in good agreement with the theoretical thermal noise, 
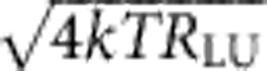
=15.7 nV Hz^−1/2^. The abrupt increase at frequencies lower than 10 Hz is generated by the power source. As expected, after applying a d.c. signal from drain to source, the low-frequency noise becomes dominated by the flicker noise. The observation that 
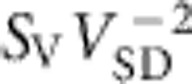
 remains almost the same from 0.1 to 2 μA at 10 Hz in Fig. 3d suggests that the mobility fluctuation attributes to the flicker noise at low frequencies according to Hooge's empirical equation^30^,^32^. At high frequencies, the flicker noise will eventually become negligible compared with the thermal noise.

Being the noise measured between terminals that are orthogonal to the input current flow, it is not evident how the flicker noise appearing in the presence of current couples to the orthogonal terminals, where in principle no current flows and why it is related to mobility fluctuations. It is possible that the finite input impedance of the noise measurement amplifier enables a small current flow through the upper and lower electrodes. Also, the device is not entirely symmetric due to the imperfection of nanolithography, making the carriers flowing into and out of the vertical terminals in different current paths and densities. Further, studies need to be carried out to understand the exact mechanism.

It is useful to note that the corner frequency *f*_c_ (the frequency at which the amount of flicker noise equals the thermal noise) can be as low as 2 kHz, which is significantly lower compared with normal detector diodes^33^. This makes it much easier in practical applications to allow the use of standard beam chopping and other simple modulation techniques without degradation of the signal-to-noise ratio.

The minimum power that a microwave sensor can detect is determined by the NEP, which is given by the measured noise divided by the responsivity. In Fig. 3a, the achieved minimum NEP is 0.64 pW Hz^−1/2^, which is more three orders magnitude better than the previous device made of low-mobility graphene on SiO_2_ without BN encapsulation^10^. Such an NEP value is even comparable to commercially available superconducting bolometers, but without the need to cool the device to cryogenic temperatures. To the best of our knowledge, this has never been achieved before at the room temperature using a solid-state device.

## Discussion

Currently, the BR still has a relatively large input resistance, *R*_SD_≈22 kΩ, as shown in Supplementary Fig. 4, which can cause significant signal reflection if coupled with high-frequency antennas. However, in principle, many layers of graphene could be stacked on top of each other separated by a thin BN spacer to reduce the overall resistance without sacrificing the carrier mobility. The planar nature of the BR means that these multi-layer devices are only marginally more complicated to fabricate. Such an architecture with parallel devices would also reduce the overall noise, hence improving the NEP figure. Further, tailored geometry and microwave strip lines can also be introduced, depending on the application to allow for better impedance matching.

To fabricate a graphene BR on an insulating substrate with a desirable carrier concentration for high-frequency applications, it is possible to use an insulating layer on top with a local gate to shift the Fermi energy. However, this will introduce some parasitic capacitance, and may hamper the high-frequency performance. Alternatively, it is possible to carry out chemical doping in various ways, such as using nitric acid^34^. It is also possible to apply a polar self-assembled monolayer such as fluoroalkylphosphonic acid at the interface to generate free carriers without an external gate^35^.

For certain applications, such as radio-astronomy or THz detectors, the cost of exfoliated devices might be acceptable. Devices based on chemical vapor deposition (CVD) graphene would significantly reduce the cost and therefore have a much wider range of applications. Currently, the carrier mobility in CVD graphene is generally much lower than exfoliated graphene, but it is improving constantly. The most recent paper by Banszerus *et al*.^36^ reported a carrier mean free path of exceeding 0.5 μm at room temperature^37^, suggesting that major progress is being made towards scalability.

In summary, a very high responsivity has been achieved at low frequency in an asymmetric cross-junction by using graphene that has a carrier mobility close to the theoretical limit. Having an intrinsic zero threshold and being nearly immune to the noise of input current due to the orthogonal arrangement of the output and input channels, the BR exhibits very low flicker noise and the low-frequency noise is mainly limited by thermal noise. The thus obtained intrinsic noise-equivalent power is found to be as low as 0.64 pW Hz^−1/2^ at room temperature, a value that is even comparable to superconducting bolometers, which however need to be cooled to cryogenic temperatures. The encapsulation of the device in BN layers also enables operation in atmospheric conditions and shows promise for a range of applications in high-frequency electronics.

## Methods

### Fabrication method

Graphene flakes for the device are fabricated using the exfoliation technique on 290 nm SiO_2_ (ref. 35) and then identified by optical contrast. They are laminated between two atomically flat and clean BN flakes by a modified stamp transfer method (modifications described in Supplementary Note 1) (ref. 1). Contacts are defined by electron beam lithography in a bilayer stack of Poly(methyl methacrylate) (PMMA) 495 K (100 nm)/950K (100 nm) and one-dimensional contacts are created by etching through the whole stack of BN–graphene–BN. The etching is done in an Oxford reactive ion etching (RIE) with CHF3 and O2 with radio frequency (RF) power of 5 W and inductively coupled plasma (ICP) power of 50 W. Three nm of chromium and 80 nm of gold are deposited using an electron beam evaporator. The same PMMA is used to lift off the remaining gold leaving the contacts. A single layer of 495 K molecular weight PMMA (100 nm) is then spun and the etch mask is defined by electron beam lithography, including both device geometry and Hall bar devices for mobility measurements. The contact resistances were measured between 0.8 and 1.4 kΩ.

### Noise measurement

The low-frequency noise was measured with a set-up based on the cross-correlation technique with two independent amplifier channels in a metal box. Each channel includes two Linear Technology 1169 op amps and is powered by batteries. The gain was set to 1,111. The output signals were sampled by an NI USB 6211 data acquisition unit at a sampling rate of 125 s^−1^. A time window of 1 s with 100 repetitions was used to measure the noise from 1 Hz to 10 kHz. The current was applied by a Keithley 2400 source meter through a 0.05 Hz low-pass filter. The set-up was tested with several resistors from 1 kΩ to 5 MΩ and the results agreed well with the theoretical values. Additional research data supporting this publication are available as Supplementary Information accompanying this publication at DOI: 10.1038/ncomms11670.

## Additional information

**How to cite this article**: Auton, G. *et al*. Graphene ballistic nano-rectifier with very high responsivity. *Nat. Commun.* 7:11670 doi: 10.1038/ncomms11670 (2016).

## Supplementary Material

Supplementary Information Supplementary Figures 1-4, Supplementary Note 1 and Supplementary References
