# Supplementary material for: Graphene ballistic nano-rectifier with very high responsivity
Source: Nat Commun. 2016 May 31;7:11670. doi: 10.1038/ncomms11670 (PMC4895026; doi:10.1038/ncomms11670)
Supplement: Supplementary Information — Supplementary Figures 1-4, Supplementary Note 1 and Supplementary References [file ncomms11670-s1.pdf]

Supplementary Figures

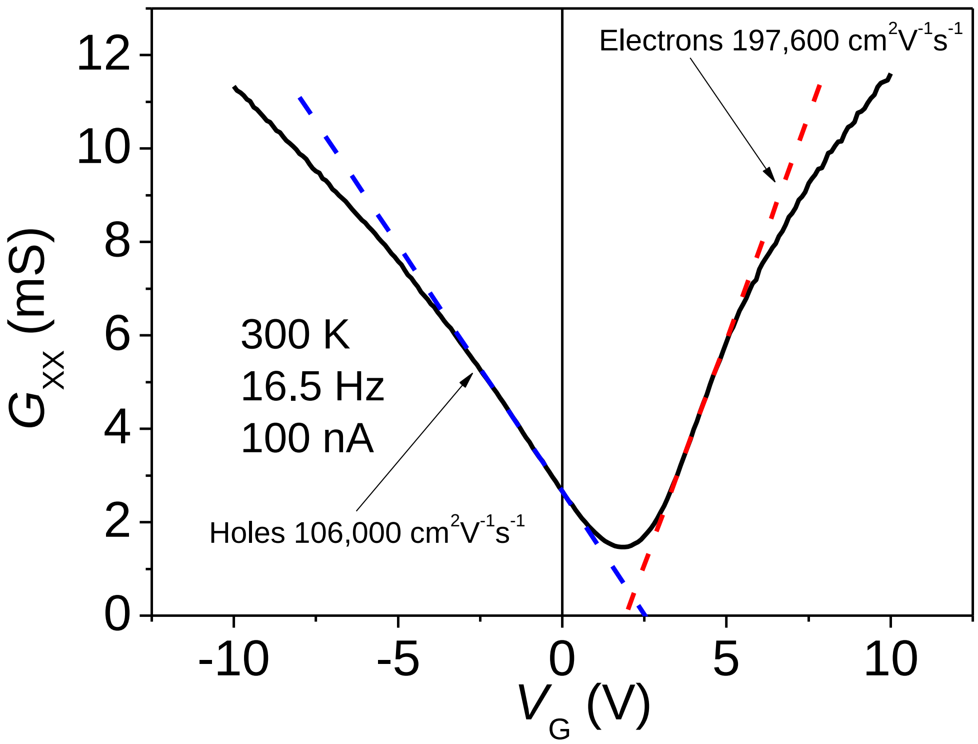

Supplementary Figure 1: Shows the Longitudinal conductivity of the graphene. The blue and red dashed lines show where the fit for the mobility was made for both the holes and the electrons, respectively.

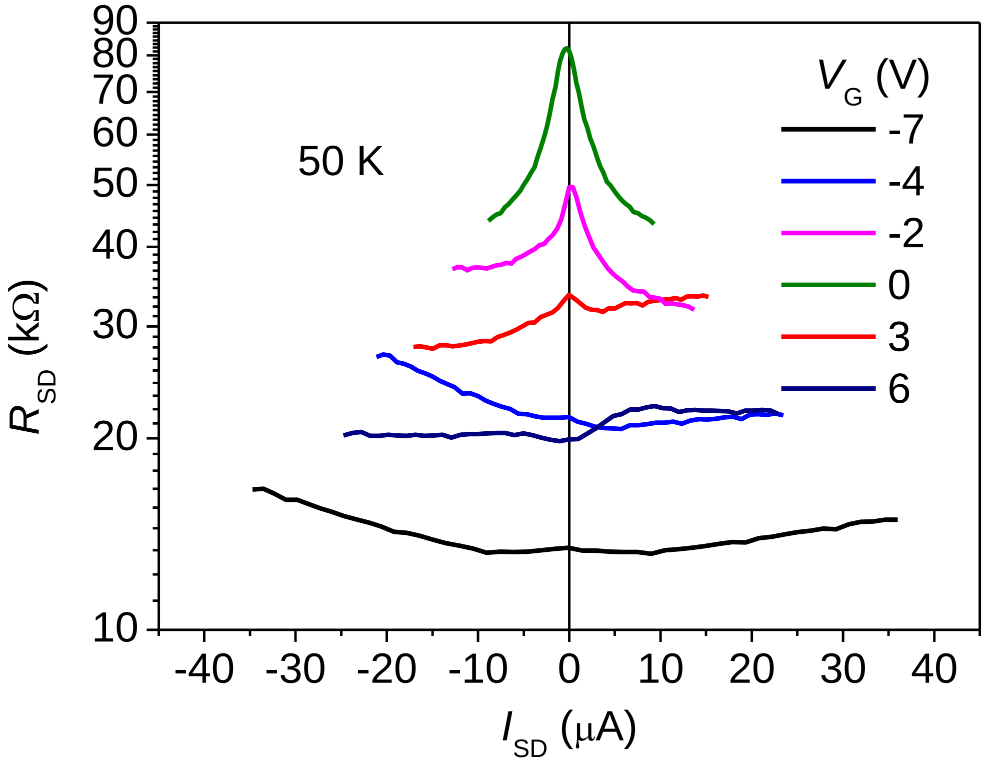

Supplementary Figure 2: Shows the resistance from S to D measured with DC in a 4-point set up as the device is measured. Clear non-linear resistance can be seen. Near the Dirac point the graphene is behaving as a semiconductor since the resistance decreases with higher current. At higher densities the graphene acts more like a metal and the resistance rises with higher current.

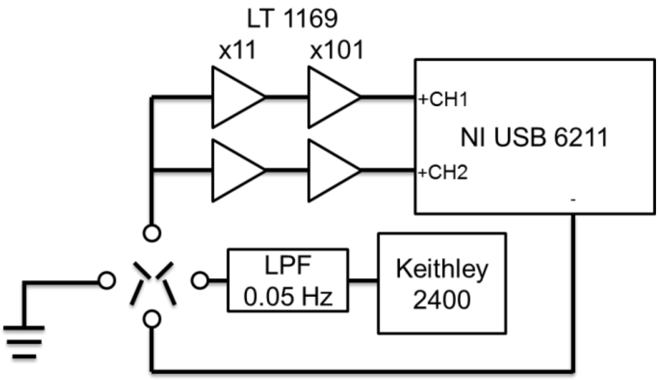

Supplementary Figure 3: Schematic diagram of the low-frequency noise measurement setup. The current was applied by a Keithley 2400 power source through a 0.05 Hz low-pass filter. The noise of the rectifier was amplified and measured by two independent channels.

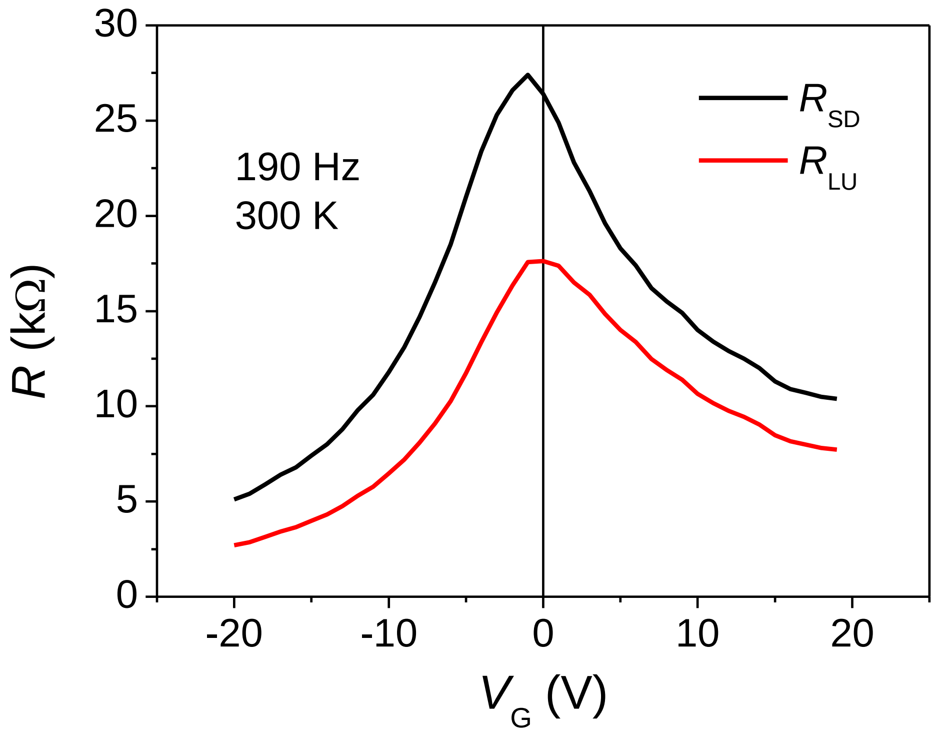

Supplementary Figure 4: Shows the resistance of the device from S to D and from L to U from 4 point AC measurements. These values where used to calculate the NEP and responsivity.

Supplementary Note 1

The stamp transfer method developed for this work is similar to the method described in ref 1 except a PMMA membrane is used to pick up the flakes. The flakes were firstly mechanically exfoliated onto the surface of 290nm SiO_2 thermally grown on a highly p-doped Si substrate. The PMMA membrane was then fabricated by initially spinning PMGI onto a Si substrate at 3000rpm with ramp 3000rpm^2 and then baked at 130°C for 3 minutes. This was allowed to cool and placed back on the spinner so that an 8% PMMA in anisole with molecular weight 950k could be spun on at the same speed and ramp as before. This was then baked at 130°C for a further 5 minutes. A clean flat area of the membrane would be chosen and a 3mm radius circle scratched into it. This would be lifted from the surface using MF319, put into water and fished onto a 2mm washer covered in dry PMMA. This membrane was baked. Both boron nitride and graphene could be picked up and stacked directly from the surface of the SiO_2 provided it was not plasma treated.

Supplementary Equation 1[2]

$$Responsivity = \frac{\frac{\partial^2 V}{\partial I^2}}{2\frac{\partial V}{\partial I}}$$

Supplementary Equation 2[3]

$$NEP = \frac{\sqrt{4kTR_{\mathrm{LU}}}}{Responsivity}$$

Supplementary References

1 Wang, L., et al. One-Dimensional Electrical Contact to a Two-Dimensional Material, Science, 342(6158): p. 614-617 (2013).

2 Kazemi, H., et al. Ultra sensitive ErAs/InAlGaAs direct detectors for millimeter wave and THz imaging applications. in Microwave Symposium, 2007. IEEE/MTT-S International. (2007).

3 Manasreh, O. *Introduction to nanomaterials and devices*. John Wiley & Sons, (2011).
